# An Additive Manufacturing MicroFactory: Overcoming Brittle Material Failure and Improving Product Performance through Tablet Micro-Structure Control for an Immediate Release Dose Form

**DOI:** 10.3390/polym16182566

**Published:** 2024-09-11

**Authors:** Elke Prasad, John Robertson, Gavin W. Halbert

**Affiliations:** 1 EPSRC Future Manufacturing Research Hub in Continuous Manufacturing and Advanced Crystallisation, University of Strathclyde, Technology and Innovation Centre, 99 George Street, Glasgow G1 1RD, UKg.w.halbert@strath.ac.uk (G.W.H.); 2 Strathclyde Institute for Pharmacy and Biomedical Sciences, University of Strathclyde, 161 Cathedral Street, Glasgow G4 0RE, UK

**Keywords:** additive manufacturing, melt extrusion, solid dosage form, oral drug delivery, solid dispersion, dissolution, formulation

## Abstract

Additive manufacturing of pharmaceutical formulations offers advanced micro-structure control of oral solid dose (OSD) forms targeting not only customised dosing of an active pharmaceutical ingredient (API) but also custom-made drug release profiles. Traditionally, material extrusion 3D printing manufacturing was performed in a two-step manufacturing process via an intermediate feedstock filament. This process was often limited in the material space due to unsuitable (brittle) material properties, which required additional time to develop complex formulations to overcome. The objective of this study was to develop an additive manufacturing MicroFactory process to produce an immediate release (IR) OSD form containing 250 mg of mefenamic acid (MFA) with consistent drug release. In this study, we present a single-step additive manufacturing process employing a novel, filament-free melt extrusion 3D printer, the MicroFactory, to successfully print a previously ‘non-printable’ brittle Soluplus^®^-based formulation of MFA, resulting in targeted IR dissolution profiles. The physico-chemical properties of 3D printed MFA-Soluplus^®^-D-sorbitol formulation was characterised by thermal analysis, Fourier Transform Infrared spectroscopy (FTIR), and X-ray Diffraction Powder (XRPD) analysis, confirming the crystalline state of mefenamic acid as polymorphic form I. Oscillatory temperature and frequency rheology sweeps were related to the processability of the formulation in the MicroFactory. 3D printed, micro-structure controlled, OSDs showed good uniformity of mass and content and exhibited an IR profile with good consistency. Fitting a mathematical model to the dissolution data correlated rate parameters and release exponents with tablet porosity. This study illustrates how additive manufacturing via melt extrusion using this MicroFactory not only streamlines the manufacturing process (one-step vs. two-step) but also enables the processing of (brittle) pharmaceutical immediate-release polymers/polymer formulations, improving and facilitating targeted in vitro drug dissolution profiles.

## 1. Introduction

### 1.1. Melt Extrusion Additive Manufacturing

Additive manufacturing, or 3D printing (3DP), of pharmaceutical formulations via commercially available filament-based melt extrusion printers has gained interest in recent years. This manufacturing technique offers advanced control of the overall size and shape, as well as the micro-structure of a tablet core, enabling the personalisation of medicines [1]. In an additive manufacturing process, an object is initially digitally designed by computer-aided design (CAD). This design forms the basis for and is translated into a layer-by-layer manufacture of printable material to form an object. Since the height of these layers can be defined, this technique affords a high spatial resolution, allowing for precise manufacture of micro-structure features on an object, such as an oral solid dose form (OSD). Micro-structure control, in turn, can permit fine-tuning of product release characteristics [1,2,3,4,5].

### 1.2. Fused Filament Fabrication (FFF)

Fused Filament Fabrication has been one of the most easily accessible material extrusion 3DP techniques for pharmaceutical applications since the required feedstock material can be manufactured via an established pharmaceutical manufacturing process, Hot-Melt Extrusion (HME). In an FFF process, the filament feedstock material is loaded into a spring-loaded pinch wheel drive gear. This gear exerts pressure on the filament, and as the gear rotates, the filament is conveyed into the hot end of the printer. Here, the filament is softened or melted. The solid part of the filament acts as a piston to extrude the softened (melted) material onto a substrate/print bed through a nozzle at the exit of the hot end.

### 1.3. Feedstock Filament Material Properties for FFF

**Figure 1:**
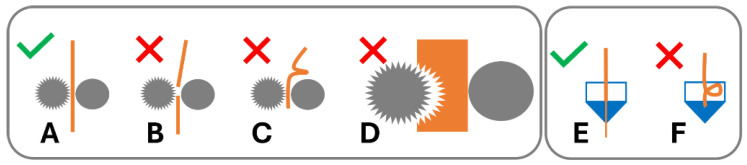
Filament performance in 3D printer drive gear and hot end. (**A**) Filament conveyed by drive gear. Failure modes in drive gear: (**B**) brittle failure, (**C**) ductile failure/buckling, and (**D**) soft filament shearing. Hot end: (**E**) material conveyed in hot end; (**F**) buckling in hot end of 3D printer.

**Figure 2:**
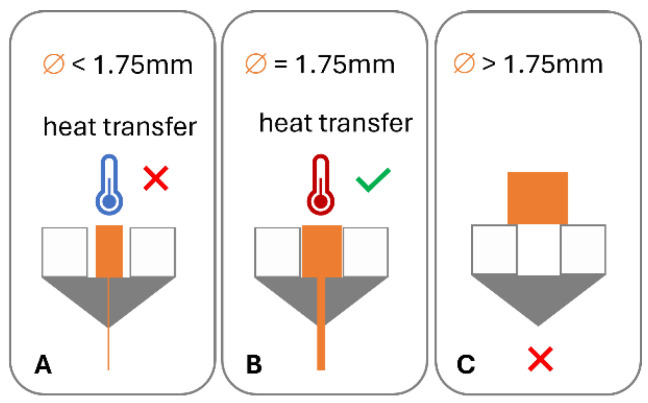
Impact of filament dimensions on heat transfer in hot end and dosing accuracy: (**A**) low diameter, poor heat transfer, and under dosing; (**B**) target diameter, good heat transfer, and accurate dosing; and (**C**) diameter in excess of hot end diameter.

In order for this process to work optimally, the feedstock material requires specific dimensional, mechanical, and rheological properties [6]. A variety of failure modes can be associated with this process. The filament may fail to be conveyed by the drive gear due to brittle feedstock material breaking [7,8,9,10,11,12] (Figure 1B) and soft material buckling [8,10,11,12,13,14,15,16] (Figure 1B) or shearing [8,15] (Figure 1C) in the drive gear. Failure of material feeding into the printer, such as buckling in the hot end (Figure 1F), may occur when the ratio of mechanical properties (flexural modulus) of the filament at room temperature to the complex viscosity at the print temperature are unfavourable [6,12,14,17]. Dimensional accuracy of the filament is also important, as deviations from the filament diameter can be associated with insufficient heat transfer in the hot end and failure to reach the ideal product temperature, impacting the material’s tenacity to the print bed and/or previously printed layers or may result in underdosing [13] (Figure 2).

### 1.4. Model Drug Mefenamic Acid (MFA)—Formulation Approaches to Improve Drug Product Performance

Mefenamic acid (MFA) is a non-steroidal anti-inflammatory drug used in the treatment of rheumatoid arthritis and menstrual disorders [18]. Mefenamic acid dose forms are commercially available as a powder-filled capsule formulation (250 mg) and a tablet formulation (500 mg). MFA is a DCS (Development Classification System) class IIa compound, exhibiting low solubility (4.18 μg/mL [19]) and high permeability, and drug absorption is thought to be dissolution rate-limited [20]. Three crystal forms have been reported for MFA, with form I reported as the most stable form [21]. A range of formulation approaches improving the low solubility and variable bioavailability of MFA have been reported, ranging from micellar solutions [22], β-cyclodextrin complexes [23], self-emulsifying drug delivery systems (SEDDSs) [24] and self-microemulsifying drug delivery systems (SMEDDSs) [25,26], and solid dispersions [7,18,27,28,29,30].

In solid dispersion formulations, the drug can be dispersed in the carrier matrix on a molecular level, in crystalline form (crystalline solid dispersion, CSD), or in amorphous form (amorphous solid dispersion, ASD) [31]. These types of formulations afford a reduction in the drug particle size to the complete minimum, as well asproviding close interaction ofdrug molecule and the carrier matrix, facilitating improved wettability and therefore potentially improved bioavailability [28,32]. Prasad et al. demonstrated how an immediate release (IR) polymer (Soluplus^®^) based CSD formulation, improved the consistency of MFA drug release [18]. The rationale behind their choice of CSD formulation was the poor glass forming ability of MFA [33] and increased stability when targeting the stable crystalline MFA form I in the formulation [21]. The low processing temperature of Soluplus^®^ was desirable since MFA exhibits high vapour pressure at elevated temperatures with a tendency to sublime, as well as phase transformation to meta stable forms [34]. D-sorbitol was added to the formulation to further reduce the processing temperature [7,18]. However, the mechanical properties of these CSD filaments were unsuitable for FFF manufacture due to their brittle nature and lack of ductility [7].

### 1.5. Solid Dispersion Formulations for FFF Applications

Solid dispersion formulations can be routinely manufactured by HME in the shape of circular filaments of a specific diameter and therefore enable access to filament based melt extrusion 3D printing of these formulations via FFF machines. Although investigations aimed to identify printability criteria for feedstock material, no generally/widely applicable criteria have been established [15,17,35]. Mechanical properties of pharmaceutically relevant polymers have been investigated to identify ideal properties for FFF applications [8,13,36,37,38,39,40,41,42]. Cellulose based polymers, e.g., hydroxypropyl methylcellulose (HPMC) [13], hydroxypropyl cellulose (HPC) [36], and ethyl cellulose (EC) [37], exhibit suitable properties to produce FFF feedstock material. These filaments exhibit high maximum stress during mechanical testing but also show ductile behaviour. Pharmaceutically approved IR polymers, such as Soluplus^®^ (polyvinyl caprolactam–polyvinyl acetate–polyethylene glycol graft copolymer) [10,42], poly-vinyl alcohol (PVA) [9], Kollidon VA 64 (copolymer of 1-vinyl-2-pyrrolidone and vinyl acetate in a ratio of 6:4 by mass) [43], and Eudragit^®^ E PO (EPO, terpolymer based on N,N-dimethyl aminoethyl methacrylate with methyl methacrylate and butyl methacrylate) [41,42], are often associated with brittle mechanical properties [10,41,44], lacking the ability to deform without breaking/failing when a load is applied, e.g., in the drive gear of an FFF printer.

### 1.6. Overcoming Brittle Filament Feedstock Material Failure in FFF

To enable the use of these pharmaceutically approved IR polymers, such as Soluplus^®^, for FFF 3DP applications, extensive formulation development is required to achieve suitable properties. Formulation approaches include the addition of plasticisers, such as triethyl citrate (TEC), D-sorbitol, and polyethylene glycol (PEG) [9,10,11], but also using complex polymer mixtures [44]. Another strategy for formulations with low dimensional stability is the addition of talc or tribasic calcium phosphate to improve the stability of printed structures [43,45]. In these formulations, excipients are added merely to effect suitable mechanical and rheological properties of the filament feedstock material, potentially reducing the drug loading and release profiles of final dose forms [6,46]. Pharmaceutical formulations aim to avoid complex formulations since multiple excipients require purchasing, testing, and certifying prior to manufacture, necessitating additional time and resources. It is not only the formulation development process that adds to the increase in resources in many instances, but also the filament manufacturing step and the associated quality checks and stability testing of an intermediate (filament) product prior to the 3D printing step [6]. This is especially crucial for hygroscopic polymers, where water acting as a secondary plasticiser may render the mechanical properties of filaments unsuitable for printing upon storage [9]. The pharmaceutical formulation space to generate simple, binary formulations for FFF 3DP applications, in particular for IR formulations, is therefore very limited.

### 1.7. Filament-Free Material Extrusion

To overcome these issues and open up the pharmaceutical formulation space, melt extrusion-based 3D printing applications are being developed. FabRx is marketing the M3DIMAKER™, a small-scale batch printer based on the principle of a single screw powder extruder, which processes powder blends into 3D printed dose forms in a single step [47,48]. Pistone et al. published a study based on a similar single screw extruder [49]. A filament free printer based on powder-filled cartridges in combination with a pneumatic piston dosing system successfully produced 3D printed dose forms by melt extrusion [46]. A Melt Extrusion Deposition (MED™) system developed by Triastek produced different dose form micro-structures in combination with different formulation compositions to target specific drug release profiles [5].

### 1.8. Aims of This Study

This work is part of the EPSRC Future Manufacturing Research HUB ‘MicroFactory’ research theme at CMAC. This project aims to implement integrated continuous, laboratory scale manufacturing platforms (‘MicroFactory’). In this work, the model drug mefenamic acid was taken through a powder blending, extrusion 3DP step to deliver optimised physical properties for targeted biopharmaceutics performance.

The aim of this study was to manufacture IR oral solid-dose forms containing 250 mg of mefenamic acid using a novel, filament free 3DP MicroFactory [6], improving the consistency in product performance compared to a commercial product, as well as creating targeted drug release profiles governed by tablet micro-structure design. This study also demonstrates how this novel technology widens the pharmaceutical formulation space for FFF applications, overcoming the (brittle) material-based limitations of FFF feedstock filaments.

## 2. Methods and Materials

### 2.1. Materials

Soluplus^®^ polymer was donated from BASF (Ludwigshafen, Germany). Mefenamic acid (MFA) (purity (HPLC) ≥ 98%), D-sorbitol Emprove Parteck^®^ SI 150 Ph Eur (purity (HPLC) 97.0–100.5%), sodium dodecyl sulphate Ph Eur (SDS), Tris(hydroxymethyl)aminomethane (Tris) (99.0–100.5% Ph Eur, USP), and trifluoroacetic acid (TFA) suitable for HPLC (purity (HPLC) ≥ 99.0%) were purchased from Sigma Aldrich (Gillingham, UK). Ethanol absolute (≥99.8%, Ph Eur) and phosphoric acid (≥85%, for HPLC) were purchased from VWR (Lutterworth, UK). A commercial mefenamic acid product, 250 mg mefenamic acid capsules, were purchased from Pharmvit Limited (PVL) (Birmingham, UK).

### 2.2. Formulation Preparation

Prior to weighing, powders were passed through a 1 mm mesh sieve. Powder samples were then mixed in a Pharmatech Bin blender AB-015 (Coleshill, UK) equipped with a 5 L vessel for 150–200 g samples. Blending was carried out at 25 rpm with an agitator speed of 100 rpm for 20 min. The powder blend contained 50% *w*/*w* mefenamic acid, 42.5% *w*/*w* Soluplus^®^ polymer, and 7.5% *w*/*w* D-sorbitol (50MFA).

### 2.3. HME-3D Printing

Hot-Melt Extrusion was performed as previously described on a Process 11 (Thermo Fisher, Karlsruhe, Germany) twin screw extruder with a length (L)-to-diameter (D) ratio of 40 ¾ equipped with a novel, custom-made (3D printed) die [6]. The screw was configured as follows: 14 feed screws −6 × 60° F bilobe mixing elements—7 × feed screws—3 × 30° F, 3 × 60° F, 4 × 90° bilobe mixing elements—13 feed screws—discharge element. The custom-made die contained a metering device which facilitated material deposition onto a print bed (Intellectual Property Office UK, patent application number 2101534.2). The metering device and print bed were controlled through a Duet 2 controller (Duet3D Ltd., Peterborough, UK). Die pressure was measured using a 2000 Series melt pressure transducer with a pressure limit of 100 bar (Terwin Instruments Ltd., Bottesford, UK). Pre-mixed powder blends were fed into the HME by means of a Brabender loss in weight (LIW) feeder machine (type DDW-N-MT) with twin concave screws (TC12/12) (Brabender, Duisburg, Germany). The feeder was calibrated for maximum output. HME process torque data are shown as % of maximum torque (12 Nm). The 3D printer bed was equipped with a Tresbro Creality 3D Printer Flexible Magnetic Hot Bed (Shenzhen, China). The printer was equipped with a round 0.4 mm diameter nozzle.

A pharmaceutically acceptable tablet shape was sought, and an elliptical tablet shape with bevelled (filleted) edges was designed with Autodesk Fusion 360 software (Version 2.0.17954). Tablet dimensions were adjusted to meet the target therapeutic dose of 250 mg MFA. An open-source slicer software, ‘Cura for Startt 1.1.1’, was used to generate stereolithography (STL) files. Linear print speeds of 5, 7.5, 10, 20, and 40 mm/s were investigated.

In order to assess the impact of tablet micro-structure on product performance, three tablet micro-structures were printed: (A) a tablet shell without top or bottom layers, (B) a tablet shell without a top layer, and (C) a tablet with a complete shell.

Tablet dimensions were measured with digital callipers (Axminster.co.uk, 0.01 mm), and the uniformity of dimensions was calculated. Tablet mass was measured using a 2DP analytical balance (Sartorius, UK), and the uniformity of mass was calculated.

### 2.4. HPLC Content Analysis

The content analysis method by HPLC was previously reported [18]. Briefly, an analysis was performed on an Agilent 1100 LC system equipped with a G1315A Diode Array Detector using a reversed-phase C-18 stationary phase (Kinetex2.6u C18, 50 × 3 mm) using (Ultra Violet) UV detection and quantification at 278 nm wavelength. A gradient method [18] was used with mobile phase (MP) A 0.5% TFA in dH_2_0 and MP B 0.5% TFA in HPLC grade Acetonitrile with an injection volume of 10 µL. Samples were analysed at 30 °C with a flow rate of 1.47 mL/min. System suitability test and bracketing standards were run. The HPLC method was validated for the presence of the Soluplus^®^-D-sorbitol polymer matrix. The method proved valid across an MFA concentration range of 50–300 µg/mL. The linearity was good with an r^2^ value of 0.9997 and recovery values ranging from 98–100.8%.

### 2.5. Rheology Analysis

Physical mixtures (PMs) of mefenamic acid, Soluplus^®^, and D-sorbitol were analysed on a Haake Mars III rotational rheometer (Thermo Fisher, Germany) equipped with a 25 mm diameter parallel plate geometry [18]. 750 mg of powdered sample were compacted under a vacuum with a compaction force of 2 tonnes for 2 min using a manual hydraulic press. 3D printed 25 mm diameter discs with a height of 1.5 mm were prepared using the filament-free 3D printer. Zero gap height calibrations were performed prior to rheological analysis. Measurements were performed in the linear visco-elastic region (LVR) of Soluplus^®^ (whereby the end of the LVR was based on 5% deviation).

Oscillatory temperature sweep: sample discs were loaded at 160 °C and equilibrated for 5 min. Temperature sweeps were performed from 160 °C to 110 °C with a constant deformation of 0.005% at a frequency of 1Hz. The gap setting was normal force controlled at 0.1 N.

### 2.6. FTIR Analysis

An FTIR analysis of raw materials, PMs, and 3DP tablets was carried out on a Bruker Tensor II equipped with a platinum attenuated total reflectance (ATR) accessory. Interferogram position and amplitude checks were performed prior to analysis. FTIR scans were performed using a KBr beam splitter with a 6 mm aperture and a 7.5 KHz scanner velocity. Samples were analysed with 16 scans at a resolution of 2 cm^−1^ and data recorded for wavenumbers in the range of 4000–400 cm^−1^.

### 2.7. Thermal Analysis: Differential Scanning Calorimetry (DSC)

Thermal analysis of PMs and 3D printed tablets was performed on a DSC214 Polyma, Netzsch (Selb, Germany). Samples were accurately weighed into 25 µL aluminium crucibles and sealed with a pierced lid. 3D printed tablets were cut prior to weighing. 5–10 mg of sample was analysed at a 20 °C/min heating rate in three cycles, (1) from 0 to 240 °C, (2) from 240 to 0 °C, and (3) from 0 to 240 °C, using helium purge gas at 40 mL/min and helium protective gas at 60 mL/min. The method used inverted, pierced lids to allow for more space for printed samples. The reference crucible was also analysed with an inverted lid.

### 2.8. X-ray Powder Diffraction Analysis (XRPD)

XRPD data were collected on a Bruker D8 Advance II diffractometer (Bruker, Germany) with the following experimental setup: For crystalline form identification, a small quantity (10–50 mg) of sample was analysed using transmission XRPD data collected on a Bruker AXS D8 Advance transmission diffractometer equipped with θ/θ geometry, with primary monochromated radiation (Cu Kα1 λ = 1.54056 Å), a Vantec position sensitive detector (PSD), and an automated multiposition x-y sample stage. Samples were mounted on a 28-position sample plate supported on a polyimide (Kapton, 7.5 µm thickness) film. Data were collected from each sample in the range of 4–35° 2θ with a 0.015° 2θ step size and a 1 s per step count time. Samples were oscillated in the x-y plane at a speed of 0.3 mm s^−1^ throughout data collection to maximise particle sampling and minimise preferred orientation effects.

### 2.9. Dissolution

Dissolution testing was performed as previously described using an ADT8i Dissolution bath (USP II, paddle) apparatus with a closed-loop setting and a T70 + UV/Visible Spectrophotometer (Automated Lab Systems, Wokingham, UK) [18]. Sink conditions during the dissolution assay were met employing a dissolution medium of 0.05 M Tris dissolution buffer at pH 9 containing 2% SDS (USP 37 Mefenamic acid capsules). A volume of 1000 mL was used to allow for in-line UV analysis. The assays were run at 50 rpm and 37 ± 0.5 °C. Samples were filtered through 0.2 μm filters prior to UV analysis (286 nm, 1 mm pathlength). Sampling was performed at 5 min intervals. The UV metric analysis was validated for the presence of excipients (Soluplus^®^, D-sorbitol) across an MFA concentration range of 100–300 μg/mL. Good linearity with r^2^ = 0.9996 and recovery values of 97.5–105.6% were observed and deemed acceptable.

Dissolution of tablet micro-structure A (Infill 47.3%, no top, no bottom layer) was performed on an ERWEKA DT 726 USP II dissolution system. Sampling was performed manually at 10 min intervals, and samples were filtered through 0.2 μm filters prior to analysis. Quantification of MFA was performed by HPLC analysis (as described above).

### 2.10. Mathematical Description—Weibull Model

A mathematical model was fitted to the experimental dissolution data in order to correlate the tablet micro-structure to in vitro drug release data. Release data were fitted using the Solver Add-in function (Version 3.0.0.1) in Microsoft^®^ Excel^®^ for Microsoft 365 MSO (Version 2408 Build 16.0.17928.20114) 64-bit. Model specific variables were selected, and constraints set while allowing for a ±5% deviation from the maximum drug release value. A normalisation factor (NF) (%) was applied to the mass fraction term, M_t_/M_∞_, to scale the accumulated drug release to percentage (%) release (rather than fractional release). The model was solved for the lowest residual sum of squares value and the model variables values were reported. The goodness of fit (r^2^) was calculated and reported for the experimental dissolution data versus modelled data using the RSQ function in Microsoft Excel for Microsoft 365 MSO (Version 2408 Build 16.0.17928.20114) 64-bit, returning the square of the Pearson product moment correlation coefficient.

The Weibull (W) model equation can be used to describe almost all release profile curves (Equation (1)). Since this is an empirical model, it does not relate to any underlying physical release mechanisms [50,51,52]:

(1) $$\frac{M_{t}}{M_{\infty }}*NF=1-\exp\left[\frac{{-\left(t-Ti\right)}^{b}}{a}\right]$$

Equation (1): Weibull model (*a*, *b*, *Ti*).

Where *M_t_* = the mass accumulated at time, *t*; *M_∞_* = the mass accumulated at infinite time, *NF* = the normalisation factor (%); *t* = time (min); *Ti* = the location parameter (lag time before the onset of dissolution); *a* = the scale parameter; and *b* = the shape parameter (release exponent).

It can also be written as

(2) $$\frac{M_{t}}{M_{\infty }}*NF=1-\exp\left[{-(kd*t)}^{n}\right]$$

Equation (2): Weibull model (*kd*, *n*).

Where *kd* (=1/*a*) is the scale parameter, and *n* is a shape parameter (release exponent) [53].

## 3. Results

### 3.1. 3D Printing of Dose Forms

The 3D printing process temperature for the 50MFA formulation was based on previous work by Prasad et al. [18], reporting 125 °C as the lowest possible HME process temperature on a small-scale twin screw extruder. However, the lowest process temperature was associated with high die pressure fluctuations. Aiming to avoid die pressure fluctuations, a slightly higher process temperature of 140 °C was chosen for this study.

The 50MFA formulation processed well when the printer was equipped with a round 0.4 mm diameter nozzle, and a process temperature of 140 °C, a linear print speed of 20 mm/s, and a layer height of 0.2 mm were selected. Elliptically shaped tablets were printed with a length of 22 mm, width of 12 mm, and height of 5 mm to meet the targeted MFA dose of 250 mg with a tablet core weight of 500 mg (±20%).

**Figure 3:**
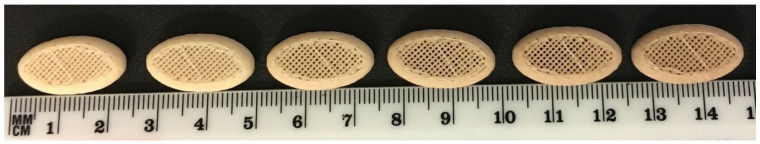
Successive prints of tablet micro-structure A (47.3% infill, no top or bottom layer) with 50MFA at 140 °C, layer height of 0.2 mm and 0.4 mm nozzle.

The visual appearance of freshly printed tablets was off-white in colour. With increasing number of prints, tablet discolouration to light brown was observed (Figure 3). In order to limit discolouration (based on visual assessment), tablets were printed in sets of six tablets, followed by purging of the system with fresh material.

**Figure 4:**
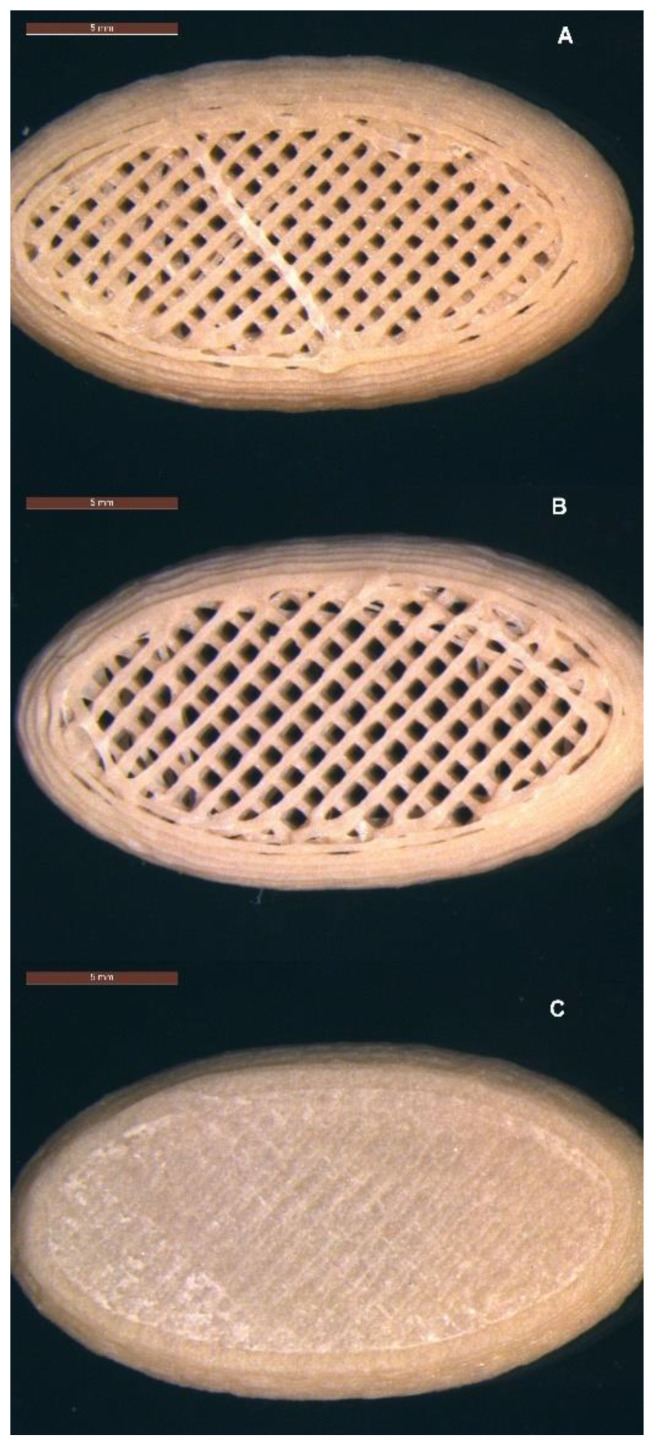
Micro-structures of 3D printed tablets containing 50% *w*/*w* MFA (22 mm × 12 mm × 5 mm): (**A**) infill 47.3%, no top, no bottom layer; (**B**) infill 40.6%, no top layer; and (**C**) infill 37.5%, top and bottom layers. Scale bar: 5 mm.

Three micro-structure designs were created: (A) infill 47.3%, no top, no bottom layer; (B) infill 40.6%, no top layer; and (C) infill 37.5%,top and bottom layers (Figure 4A–C). The distance between the individual infill lines was calculated by the ‘Cura for Startt 1.1.1’ slicer software as 0.85 mm for structure A, 0.99 mm for structure B, and 1.14 mm for structure C (Table 1). The surface area of the tablet shell and the volume of the tablet were determined using Autodesk Fusion 360 software. The estimated surface area (SA) of the pores was calculated based on the assumption of an ideal square pore with each of the four sides of the square pore calculated by distance between infill lines multiplied by the tablet height (5 mm). The number of pores initially accessible to the dissolution medium was manually determined by counting the pores in optical images. The surface area-to-volume ratio was calculated and is shown in Table 1.

The printed sets of six tablets showed good uniformity of mass (Table 2), complying with pharmacopeial requirements. The percentage difference in the maximum and minimum weight from the average were −1.0% and −2.2%, respectively.

### 3.2. HME Process Parameters

**Figure 5:**
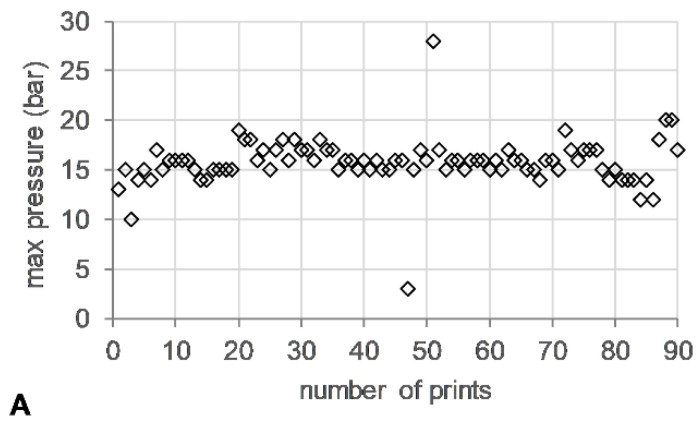

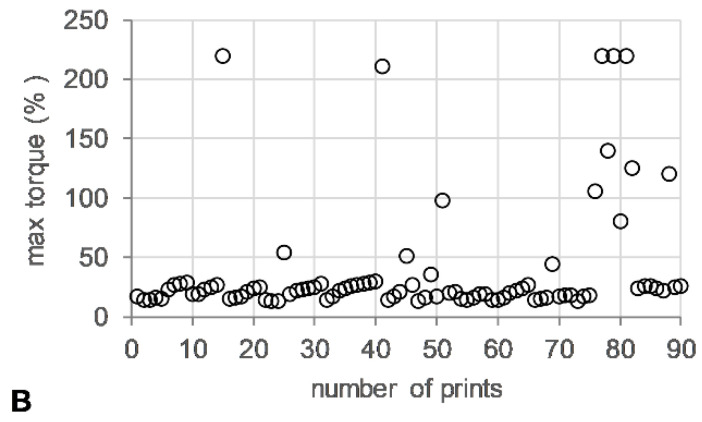
HME-3DP process data. (**A**) Maximum die pressure (bar) (open diamonds) and (**B**) maximum torque (%) (open circle) versus number of prints.

The die pressure recorded during the operation of the HME, used as an indicator for the level of fill of the metering device, remained stable (~15 bar) (Figure 5A). Torque values ranged from 13–24%, with short spikes of up to 200% associated with start/stop events of the 3DP process (Figure 5B).

### 3.3. HPLC Content

The uniformity of content was determined by analysing the endpoint of the dissolution assay, with a content of 48.3% ± 0.3% *w*/*w* MFA (n = 6). This was in agreement with previous studies suggesting a loss of MFA through sublimation when processed at elevated temperatures [7,18].

### 3.4. Rheology

**Figure 6:**
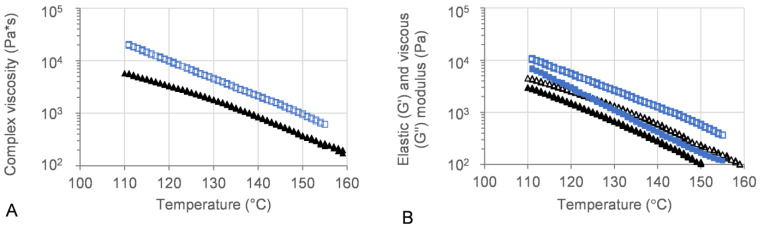
Oscillatory temperature sweep of 50MFA-SOL. (**A**) Complex viscosity and (**B**) storage (G′) and loss (G″) modulus vs. temperature. Storage modulus: filled; loss modulus: open. 3D printed discs (140 °C) shown in blue, 50MFA extrudate processed at 125 °C [7] shown in black.

Oscillatory temperature sweeps were performed for 50MFA 3D printed discs, with the complex viscosity ranging from 10^2^ Pa·s at 155 °C to 10^4^ Pa·s at 110 °C (Figure 6A, blue squares and triangles). These values are within the ideal complex viscosity values of 8 × 10^2^ to 10^4^ Pa·s, reported as suitable for extrusion on small-scale extruders [54].

The viscous (G′) and elastic (G″) modulus recorded for 3D printed discs showed dominating viscous behaviour across the entire temperature range (Figure 6B). At the 3D printing process temperature, 140 °C, the complex viscosity ranged from 1.6 × 10^3^ to 2.4 × 10^3^, which proved ideal for use with the filament-free 3D printer.

### 3.5. FTIR

FTIR spectra for printed tablets confirmed the presence of mefenamic acid form I, seen as the characteristic N-H stretch of MFA form I at 3309 cm^−1^ [55]. MFA form II (N-H stretch at 3353 cm^−1^) was not detected in the printed tablets (Figures S1 and S2).

### 3.6. DSC

**Figure 7:**
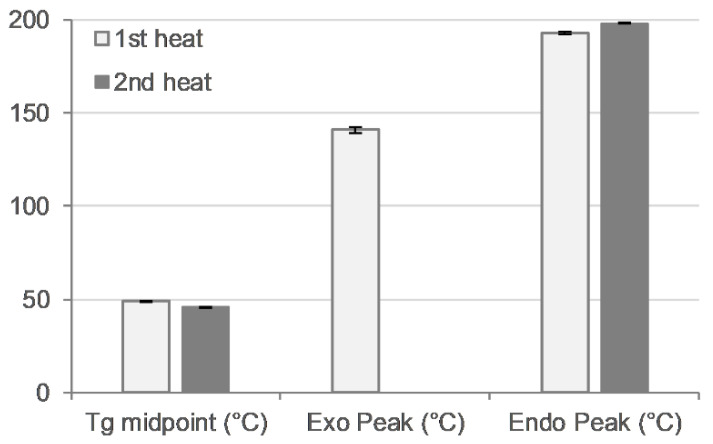
Thermal analysis of 3D printed disc. Glass transition midpoint (Tg, °C), exothermic peak (°C), and endothermic peak (°C) values for first (light bars) and second (dark bars) heating cycle (n = 2).

**Figure 8:**
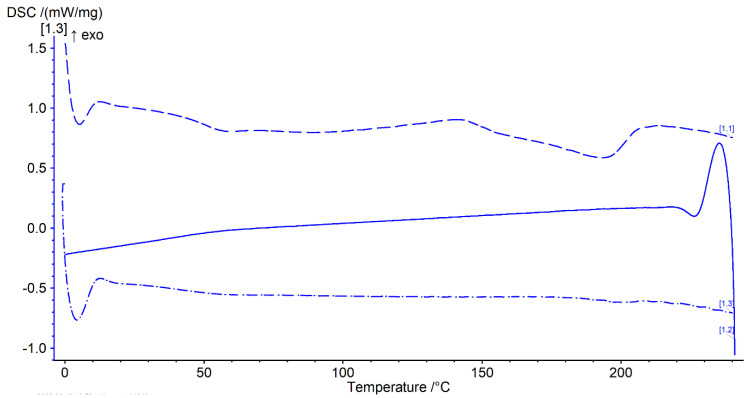
Thermogram of 3D printed discs. Top dashed lines: first heating cycle from 0 to 250 °C; middle solid line: cooling cycle from 250 to 0 °C; and bottom dash-dotted line: second heating cycle from 0 to 250 °C (all at rate of 20 °C/min) (n = 2).

Thermal analysis was performed with a heat–cool–heat cycle. In the first heating cycle, a single glass transition (Tg) at ~49 °C was followed by a broad exotherm between 139–142 °C and a melt endotherm in the range of ~196–198 °C (Figure 7 and Figure 8 top). The broad exotherm may relate to the heating (energy) induced crystallisation of supersaturated MFA in the polymer matrix, with the crystalline MFA melting in the following endotherm. In the second heating cycle, a single Tg was observed only, indicative of the absence of crystalline material (Figure 7 and Figure 8 bottom).

### 3.7. X-ray Powder Diffraction Analysis: XRPD

**Figure 9:**
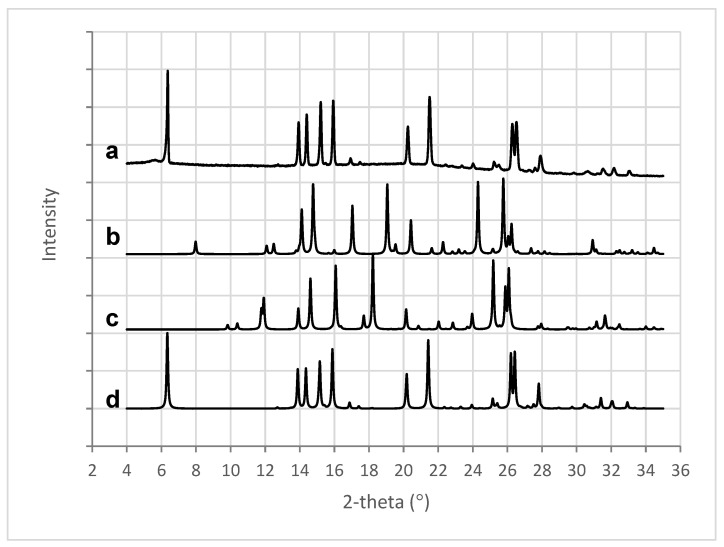
XRPD patterns for (a) 3D printed tablet containing 50% *w*/*w* MFA, (b) MFA form III [56], (c) MFA form II [57], and (d) MFA form I [58].

X-ray analysis of the 3D printed tablets showed the presence of mefenamic acid form I only, seen in the characteristic four peaks between 13° and 16° 2-theta (Figure 9a,d). Characteristic peaks for mefenamic acid form II (Figure 9c) at 11.9° and 18.2° 2-theta or form III (Figure 9b) at 17°, 19°, and 24.3° 2-theta were not observed in the 3D printed tablets.

### 3.8. Dissolution USP II

**Figure 10:**
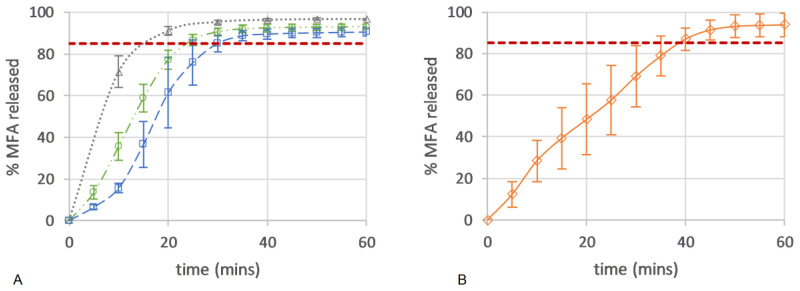
Drug release (%) of (**A**) 50MFA 3D printed tablets (n = 6) over time with different tablet micro-structures: ‘tablet A’—infill 47.3%, no top or bottom layer (grey triangle); ‘tablet B’—infill 40.6%, no top layer (green circle); and ‘tablet C’—infill 37.5%, top and bottom layer (blue square). (**B**) Powder-filled capsule with 250 mg MFA (n = 6) (Pharmvit Limited (PVL), Batch 4348) [18]. Red dashed line: 85% MFA released.

Here, the drug release from all micro-structures of the 3D printed dose forms conformed to an IR profile (>85% at 45 min), with complete release seen as an endpoint in the flattening of the drug release curve (Figure 10A). All 3D printed tablets showed improved consistency in drug release with lower variability when compared to the in vitro drug release of a commercial 250 mg MFA powder fill formulation (Pharmvit Limited (PVL), Batch 4348), showing high variability in the standard deviation (up to 17%) (Figure 10B) [18]. The ability to control the spatial resolution of the tablet core design in this study further improved and enabled the manufacture of targeted IR profiles. As expected, tablet structure A, with the inner core of the tablet fully exposed to the dissolution medium (no top or bottom layer were printed; Figure 4A), showed the fastest drug release, reaching complete release at 20 min. The dissolution test of tablet structure A was analysed by a more sensitive analytical method (HPLC) in contrast to (an in-line UV analysis for) tablet structures B and C and may explain the discrepancy between the % of MFA released at the plateau/endpoint of the release curve. Changing the porosity of the core of the tablet, by distributing more material to the shell of the tablet, slowed down drug release by reducing the availability of the pores on the surface to the dissolution medium. In tablet shapes B and C, the addition of a bottom layer re-distributed material from the infill to the shell, increasing the gap between infill lines from 0.85 mm (shape A) to 0.99 mm for shape B and 1.14 mm for shape C. By closing one side of the tablet’s porous core (shape B), the hydrodynamics in the core of the tablet were significantly changed, slowing down drug release (Figure 4B), achieving complete release at 30 min. Completely encasing the porous core (shape C) in a closed shell structure reduced the interaction of the dissolution medium with the internal core of the tablet, facilitating a short delay (lag) in drug release (0–10 min). The completely sealed shell around the porous tablet core (tablet micro-structure C, Figure 4C) resulted in complete drug release at 35 min.

Applying the difference (f1) and similarity (f2) factor method [59] to the resulting dissolution profiles showed that all three 3D printed tablets were different compared to the commercial powder filled capsule product [18]. The 3D printed tablets showed a high f1 (>15) difference value, exceeding the criteria for difference (≤15), and an f2 (similarity) factor below the sameness level (50–100) (Table 3).

Comparing the 3D printed micro-structures showed that tablets A and B were similar, but tablet C was different (Table 4) with high difference and low similarity factors.

### 3.9. Mathematical Description of Dissolution Data—Weibull Model

**Figure 11:**
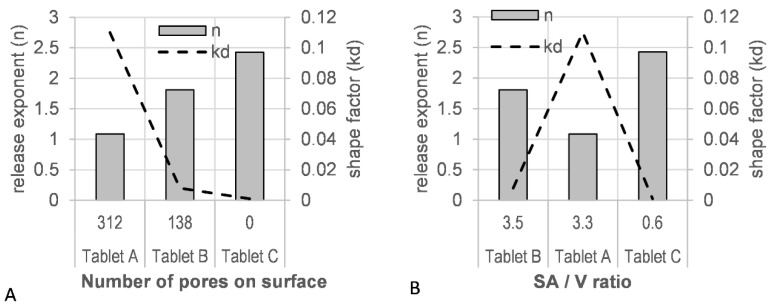
(**A**) Tablet porosity and (**B**) surface area-to-volume ratio (SA/V) vs. model parameters (release exponent, *n*; shape parameter, *kd*).

A good fit was observed for the Weibull model with a high r^2^ value (>0.9990) and a low residual sum of squares (RSS) value (<15) (Table S7). A correlation of the tablet porosity with the model derived scale factor (*kd*), as well as the release factor (*n*), was observed (Figure 11A). The level of porosity exhibited an inverse relationship to the release exponent (*n*), whereas the *kd* factor was directly proportional: the most ‘open’, porous tablet (A), with the highest number of pores initially available to the dissolution medium, showed the lowest release exponent (*n* = 1.088) and the highest shape factor (*kd* = 0.110). A correlation of the estimated SA/V with these shape and scale factors was not observed (Figure 11B).

## 4. Discussion

The objectives of this study were to overcome the brittle material failure of pharmaceutical IR polymers in FFF 3D printing and to manufacture IR tablets containing 250 mg MFA using a novel, filament-free 3D printing MicroFactory [6] with micro-structure-controlled product performance. The aim of this study was to demonstrate how this novel technology widens the pharmaceutical formulation space for melt extrusion applications, overcoming materialbased limitations of melt extrusion feedstock filaments.

In this study, a novel filament-free 3D printer was used to 3D print tablets with a pharmaceutically approved IR polymer, Soluplus^®^, formulation. Although Soluplus^®^ exhibits excellent rheological properties for HME and 3D printing applications [10,16,54,60,61], it is, as an IR polymer, very brittle in nature and therefore exhibits unsuitable mechanical properties for processing via commercial filament based melt extrusion (FFF) printers [7,9,38,41,44,62,63]. In addition, Soluplus^®^ failed to form filaments at 30% *w*/*w* PCM drug loading when extruded at 140 °C [8,64].

In line with the difficulty of developing simple, binary Soluplus^®^ based filaments for extrusion, was a recent study reporting brittle mechanical properties of a 50MFA formulation (similar to the formulation used in this study) [18]. Recently, the filament free 3D printer used in this study demonstrated the successful fabrication of 3D printed tablets [6] with a ‘non-printable’ filament (ductile filament failure) formulation in a conventional FFF printer [13]. In this study, the novel printer (MicroFactory) enabled the processing of this 50MFA formulation in a single manufacturing step without the need for formulation modifications to overcome the brittle nature of the Soluplus^®^ polymer.

Here, we demonstrated the production of highly controlled micro-structures (Figure 4) with excellent uniformity of mass and dimensions (Table 2 and Tables S1–S6) that are well within pharmaceutical specifications. During the single (streamlined) manufacturing step, discolouration of tablets was observed over time (Figure 3). A manufacturing protocol was established, purging fresh material after a fixed number of printed tablets to manage this observation. Further investigations analysing discolouration and polymer stability during processing would be interesting, but were outwith of the scope of this study. Recent reports linking Volatile Organic Compound Analysis (VOCA) by selected-ion flow-tube mass spectrometry (SIFT-MS) with thermogravimetric analysis (TGA) and HME processes were able to link process conditions to changes in VOCA products [65,66], and may be a suitable means of investigating the discolouration of tablets further. The uniformity of content of produced tablets was good, with a slightly decreased MFA content (Section 3.3). This was not surprising since MFA exhibits a high vapour pressure at elevated temperatures with a tendency to sublime [34]. A reduction in the MFA content of the same 50MFA formulation after a Hot-Melt Extrusion process was previously reported [18].

MFA powder is also known to phase transition from stable form I to metastable form II at elevated temperatures [34]. The processed material was therefore investigated for MFA polymorphic form changes, with MFA being confirmed as the stable polymorphic form I by FTIR (Figures S1 and S2) and XRPD (Figure 9) analysis. This was in agreement with previous findings of MFA remaining in the stable polymorphic form I after the Hot-Melt Extrusion process [7,30]. MFA was not only detected as crystalline form I: thermal analysis of the 3D printed tablets also indicated the presence of supersaturated amorphous MFA in the system (Figure 7 and Figure 8). This was not surprising, as previous studies have measured the equilibrium solubility of MFA in a Soluplus^®^-D-sorbitol polymer matrix at much lower (~10% *w*/*w*) drug loadings [7,67]. The Tg relating to the plasticised polymer system and the presence of crystalline MFA, were in agreement with previous studies of this formulation [18].

The rheological assessment of 3D printed discs revealed complex viscosity values in a range ideal for extrusion on small-scale extruders [54] and proved ideal for processing on the filament-free 3D printer (Section 3.4). The trend of complex viscosity across the investigated temperature range agreed with previous oscillatory temperature sweep studies of 50MFA pelletised extrudate [7]. Although the HME process conditions for 3D printed (this study) and pelletised (previous study) materials were similar in terms of the screw mixing profile, screw speed, and powder feed rate, the pelletised material was processed at a significantly lower temperature (125 °C) compared to the 3D printed discs (140 °C), very likely resulting in a higher crystalline content in the pelletised extrudates. However, the complex viscosity values for the pelletised extrudates (with a higher crystalline content) were lower than that of the 3D printed discs. This was in contrast to previous findings where higher drug loadings and crystalline content were associated with higher complex viscosity values, with the crystalline content acting like a solid filler [7,68,69]. However, it may be attributed to the higher start temperature (160 °C) of the oscillatory temperature sweep studies, facilitating MFA to dissolve in the polymer matrix. This is not unusual, Solanki et al. showed that the complex viscosity values of Itraconazole-Soluplus^®^ mixtures changed depending on the start temperature of the assay [68], despite Itraconazole not mixing well with Soluplus^®^ in the absence of the mechanical mixing profile of the HME. This was in line with previous reports of MFA requiring the mechanical mixing input of the Hot-Melt Extrusion to form a homogenous system with Soluplus^®^ [18,70]. It may also be possible that extrusion and 3D printing at higher temperatures (140 °C) generated a polydisperse system with molecularly dispersed MFA as well as distinct crystalline and amorphous regions of MFA within the polymer matrix. Thermal analysis of 3D printed discs showed exothermic crystallisation peaks, indicative of a supersaturated amorphous phase in the system (Figure 8). Recent studies relating HME process temperature to solid state changes of MFA (in a range of MFA-Soluplus^®^-D-sorbitol polymer formulations) support the observations made in this study: Vivattanaseth et al. used in-situ THz Raman analysis during HME processing, and employed Multivariate Curve Resolution (MCR) to quantify transition of crystalline MFA (form I) to an amorphous form [67]. Further studies are required to characterise the amorphous and crystalline content of the 3D printed material. The same trend was observed when assessing the viscous (G′) and elastic (G″) modulus of 3D printed discs and 50MFA extrudate, with dominating viscous behaviour observed across the entire temperature range (Figure 6B). At the 3D printing process temperature, the complex viscosity ranged from 1.6 × 10^3^ to 2.1 × 10^3^ Pa·s, which proved ideal for use with the filament free 3D printer. The viscosity range of the Soluplus^®^ based formulation was lower than a previously 3D printed, HPMC based formulation (1.9 and 6.1 × 10^4^ Pa∙s at 165 °C and 145 °C) [6]. In order to successfully process the higher viscosity material, different printing process parameters were required (Table 5). This was reflected in the maximum attainable printing speed and minimum layer heights: 50MFA was processed well at a higher printing speed with a maximum speed of 40 mm/s and a minimum layer height of 0.2 mm, whereas the HPMC based formulation only printed well at a maximum speed of 20 mm/s and a minimum layer height of 0.3 mm [6] (Table 5).

Based on Kolter’s recommendation regarding ideal viscosity values for processing polymer-based formulations on a small-scale extruder, and the complex viscosity of 50MFA at the process temperature, an operating window from ~120 °C to ~160 °C would apply to this formulation. Due to the low solubility of MFA in the polymer, this would allow for flexible manufacturing of 3D printed dose forms regarding the solid state of the API: producing mainly crystalline systems at lower process temperatures [18] and predominantly amorphous systems at higher processing temperatures [67]. This flexibility would be limited with respect to APIs exhibiting high solubility in the polymer system, such as 30 PCM-Affinisol™ 15LV [6].

In this study, we successfully produced IR 3D printed tablets containing 250 mg of MFA, exhibiting a faster and more consistent dissolution profile compared to a commercial product [18]. Although the formulation investigated in this study retained MFA, a weak, hydrophobic acid (pKa 4.2, logP 5.1, [71]), in its crystalline form, the polymer matrix facilitated excellent wettability of MFA, resulting in an IR profile of all tablet micro-structures (>85% at 45 min, Figure 10). As a non-ionic amphiphilic polymer, Soluplus^®^ has shown to improve dissolution performance facilitated via increased wettability in solid dispersion formulations in numerous studies [72,73,74,75,76,77,78,79]. The improved dissolution profiles were also related to a reduction in the surface tension of the aqueous dissolution media [78,80].

The Weibull model, a purely mathematical model, was employed to describe the product performance of tablets A–C (Figure 10) with the aim of correlating the tablet micro-structure to model parameters. The release profile with the highest number of pores (tablet A) resulted in high rate parameters (*kd*) and low release exponents (*n*) in the fitted model (Figure 11). The lowest number of pores (tablet C) was associated with low rate parameters (*kd*) and high release exponents (*n*). A good fit was observed for the Weibull model (Table S7), which, as a purely mathematical equation, does not describe the type of release mechanism or tablet geometry. Other dissolution models, such as the Korsmeyer–Peppas and Peppas–Sahlin models, aim to describe the type of release mechanism with the release exponent as Fickian diffusion, anomalous, Case-II, and Super Case-II transport [50,51,81]. However, for these models to be valid, very specific geometrical requirements need to be met, and a sufficient number of datapoints (<60% drug release) are required. Tablet structures investigated in this study, elliptical tablets with rounded edges and top and bottom layers containing multiple pores, differed greatly from these geometrical requirements. In addition, the insufficient number of datapoints available for tablet A would also result in overfitted results.

Additive manufacturing of pharmaceuticals enables the design of tablet geometries with defined geometries and tablet core designs, targeting specific surface area-to-volume (SA/V) ratios. For 3D printed tablets, it has been reported that the SA/V ratio can control the dissolution profile. In fact, many studies relating the in vitro dissolution testing of 3D printed (polymer matrix-based) dose forms to tablet design established that certain geometries with larger SA/V ratios resulted in faster drug release [82,83,84,85,86]. The Higuchi dissolution model modified by Lapidus and Lordi (for swellable matrices) describes drug release from an inert matrix via diffusion, which is directly related to the SA/V ratio of the dose form [87]. This model showed a poor fit for all 3D printed tablet structures, which may be due to the model’s assumption that drug release is solely based on Fick’s law.

Whilst the theory of SA/V playing a key role in drug release from 3D printed tablets in this study holds true for tablet A (SA/V = 3.3 mm^−1^) compared to tablet C (SA/V = 0.6 mm^−1^), tablet B does not fall into this pattern. Despite the highest SA/V ratio of tablet B (SA/V = 3.5 mm^−1^, Table 1), drug release was slower than the release of tablet structure A (Figure 10). This may be due to the different pore structures of these tablets: the pores in tablet A are open at both sides (top and bottom) of the tablet, whilst the pores in tablet B are only open on one side. The presence of pores on the tablet surface (with high porosity > 20%) has previously shown to affect the dissolution rate by creating turbulences in the hydrodynamic flow of the dissolution media, reducing the thickness of the hydrodynamic boundary layer [88]. Interestingly, only the pore diameter proved critical in the study, opposed to the available (wetted) inner surface and the pore depth-to-diameter ratio. Turbulences only arose when the pore diameter was large enough in relation to the hydrodynamic boundary layer thickness, causing increased erosion near the pore boundary on the tablet surface [88]. With pores being present on both sides of tablet A, but only on one side of tablet B, increased hydrodynamic turbulences on the surface of tablet A may have resulted in a decrease in the boundary layer thickness, effecting increased erosion near the pore boundary on the tablet surface, resulting in faster drug release. The Hopfenberg dissolution model describes drug release from various geometries via erosion only, where release is not affected by diffusion [50,51,89]. Fitting this model to the dissolution data of 3D printed dose forms showed that the erosion rate constant for tablet A was significantly higher than that of the other structures (further studies are required to consolidate these results) (Table S8). Other studies have linked higher porosity of printed tablet structures (lower infill %) to faster drug release [90,91], which is very likely due to a combination of available SA/V ratio and large pores giving rise to hydrodynamic turbulences and a reduced boundary layer thickness. The requirement for a minimum pore size may also be applicable in the case of 3D printed dose forms, where studies reported reduced drug release with a higher infill % (smaller pores) of 3D printed tablets. However, authors related this observation to entrapped air within the pores delaying the wetting of the tablets and therefore reducing the drug release rate [84,86].

Drug release from Soluplus^®^ solid dispersions has been described to follow a range of mechanisms: Fickian diffusion (Simvastatin) [92], swelling of the polymer matrix (Carvedilol) [93], as well as a combination of diffusion, relaxation, and erosion mechanisms (Sulfamethoxazole) [94]. It may therefore be possible that 3D printing OSDs may offer the ability to design tablet micro-structures targeting and resulting in specific drug release mechanisms. Further studies are required to investigate this in more detail.

The above-described differences in tablet structures are reflected in the results from the f1 difference and f2 similarity method, depicting tablet shapes A and B as similar but depicting both as different to tablet shape C (Table 6). Interestingly, when compared with the CSD formulation (pelletised extrudate presented in hard gelatine capsule) previously investigated by Prasad et al., only tablet shape A (not B or C) was deemed different (Table 6). The presentation of the pelletised extrudates in a random fashion within the hard gelatine capsule when presented to the dissolution media made it impossible to estimate the available surface area or the porosity of pellets within the capsule. A comparison based on structural features is therefore not possible but indicates that even small differences in structural features can produce different release profiles.

Conventional FFF printers work with filament diameters of 1.75 mm and 2.85 mm, with print speeds ranging from 3 to up to 90 mm/s [6], but generally a standard print speed in the range of 40–60 mm/s is used. The maximum speed to print small objects, such as pharmaceutically relevant tablets, cannot exceed 90 mm/s because the printer fails to accelerate to a higher speed at such small distances [95]. Ordinarily, the linear print speed is reported in 3D printing studies, but the volumetric flow speed provides a better depiction of the material throughput in a printing process. In this study, a linear print speed of up to 40 mm/s was achieved with the 50MFA formulation, which is equivalent to a volumetric speed of 739 mm^3^/s, signifying a substantially higher material throughput compared to a conventional FFF printer (using a 1.75 mm diameter filament) (40 mm/s ~96 mm^3^/min) [6].

The MicroFactory employed in this study offered a fully customizable screw configuration (mixing profile) and extruder shaft length with eight individually controlled temperature zones for a small-scale, twin screw hot-melt extruder (in line with a custom-made 3D printer interface), therefore allowing for a high level of customisation and a wide range of process conditions, offering the possibility to work with a wider range of pharmaceutical formulations compared to filament based 3D printers. Twin screw extruders, like the MicroFactory, have proven more efficient in providing homogeneous mixing of different ingredients compared to single screw extruders [96]. Since the MicroFactory is operated in a continuous mode, it also offers great flexibility regarding batch sizes [6].

The advantages of filament-free melt extrusion 3D printing with the MicroFactory are manifold. This case study demonstrates how a non-printable, brittle feedstock filament formulation was successfully processed on the MicroFactory in a streamlined, single manufacturing step.

This study also relates material properties to MicroFactory process parameters and relates the OSD micro-structure of a 50MFA CSD formulation to drug product performance.

## 5. Conclusions

In this study, we successfully 3D printed OSDs containing 250 mg of MFA with an IR profile and demonstrated how a filament-free HME 3D printer opens up the pharmaceutical formulation space for additive manufacturing, particularly for pharmaceutically approved IR polymers, which tend to exhibit brittle material properties.

A single-step, additive manufacturing (melt extrusion) process in the presented MicroFactory not only resulted in a streamlined manufacturing process, but also reduced time and resources in formulation development efforts. Manufactured tablets showed good uniformity of mass, dimensions and content of uniformity, complying with pharmaceutical specifications.

High spatial control over the manufacture of the micro-structure of the tablet core enabled fine-tuning of customised IR drug release profiles.

Fitting a mathematical model to the dissolution data correlated high tablet porosity (low infill %) in the tablet micro-structure with high rate parameters and low release exponents. Low tablet porosity (high infill %) was correlated with low rate parameters and high release exponents.

Whilst previous studies have attributed the drug release kinetics of 3D printed tablets to the SA/V ratio, this study has shown that the porous micro-structure of the tablet may have a greater effect on dissolution kinetics by giving rise to hydrodynamic turbulences at the surface of the tablet, reducing the boundary layer thickness.

This illustrates how the MicroFactory, presented in this study, can streamline an additive manufacturing process in a single manufacturing step, producing tablets with a defined mass, content, micro-structure and improved release properties.

This work forms part of the broader aim of the EPSRC Future Manufacturing Research HUB at CMAC. This project aims to implement integrated continuous, laboratory scale manufacturing platforms by means of crystal engineering of a model drug (MFA) coupled with polymer processing steps to deliver enhanced physical properties for biopharmaceutics performance. It forms the basis for future work within the HUB, showing how coupling crystal engineering with polymer processing may facilitate future performance-based design and the continuous manufacture of structured particulate products [7,18].

## Figures and Tables

**Table 1:** Tablet micro-structure properties: infill %, estimated pore, shell and estimated total surface area (SA), surface area-to-volume (SA/V) ratio, and number of large pores on top and bottom of tablet.

Tablet Structure	Infill %	SA/Pore (mm^2^)	Total SA Pores (mm^2^)	SA Shell (mm^2^)	Total SA (mm^2^)	SA/V (mm^−1^)	Number of Large Pores
A	47.3	17.0	2652.0	370.1	3022.1	3.3	312
B	40.6	19.8	2732.4	464.6	3197.0	3.5	138
C	35.0	22.8	0	559.0	559.0	0.6	0

**Table 2:** Mass and dimensional accuracy of tablet micro-structures (n = 6). Relative standard deviation = % RSD.

Microstructure Infill %	A 47.3%	B 40.6%	C 35.0%
Weight variation (% RSD)	0.9	0.6	1.1
Max % difference from average weight	1.7	−1.0	−2.2
Uniformity of mass	PASS	PASS	PASS
Length variation (% RSD)	0.73	0.13	0.12
Width variation (% RSD)	0.13	0.29	0.45
Height variation (% RSD)	0.49	1.39	0.99

**Table 3:** Difference (f1) and similarity (f2) factor method comparing in vitro dissolution profiles of commercial capsule product from Pharmvit Ltd. with 3D printed tablet shapes A, B, and C.

Tablet Shape	f1 Difference Factor	f2 Similarity Factor
A	28.6	41.9
B	43.4	39.1
C	23.6	52.0
limits	0–15	50–100

**Table 4:** Difference (f1) and similarity (f2) factor method comparing in vitro dissolution profiles of 3D printed tablet shapes A, B, and C.

	Tablet A vs. B	Tablet A vs. C	Tablet B vs. C
f1 (0–15)	12.7	24.7	15.4
f2 (50–100)	55.0	41.1	49.6

**Table 5:** Formulation- and temperature specific material properties (complex viscosity) and 3D printing process parameters (maximum print speed and minimum layer height): 50MFA and 30% *w*/*w* Paracetamol, HPMC (Affinisol 15LV).

Formulation	Process Temperature	Complex Viscosity (Pa∙s)	Max. Print Speed (mm/s)	Min. Layer Height (mm)
50MFA	140 °C	2 × 10^3^	40	0.2
30% *w*/*w* Paracetamol, HPMC (Affinisol 15LV)	145 °C	6.1 × 10^4^	20	0.4
	165 °C	1.9 × 10^4^	20	0.3

**Table 6:** Difference (f1) and similarity (f2) factor method comparing in vitro dissolution of 3D printed tablet shapes A, B, and C vs. pelletised MFA extrudate from [18]. Dissolution profiles are considered similar with f1 values between 0 and 15 and f2 values between 50 and 100.

	f1 Similarity Factor (0–15)	f2 Difference Factor (50–100)
Tablet A vs. B	12.7	55.0
Tablet A vs. C	24.7	41.1
Tablet B vs. C	15.4	49.6
Extrudate vs. A	21.6	48.4
Extrudate vs. B	7.6	66.0
Extrudate vs. C	9.4	60.2

## Data Availability

All data underpinning this publication are openly available from the University of Strathclyde KnowledgeBase at https://doi.org/10.15129/880df3f3-bb94-4e14-9802-cc61ae9689e3.

## Supplementary Materials

The following supporting information can be downloaded at: https://www.mdpi.com/article/10.3390/polym16182566/s1. Figure S1: FTIR spectra of MFA: Form I (blue) with N-H stretch at 3307 cm^−1^, Form II (red) with N-H stretch at 3344 cm^−1^. Figure S2: FTIR spectra of MFA form I (blue), form II (red), 3DP tablet (green), Soluplus^®^ (olive) and D-Sorbitol (orange). Table S1: Uniformity of mass tablet structure A: weight (mg), average weight, standard deviation (stdev), % relative standard deviation (%RSD) and % Deviation from average weight. Table S2: Uniformity of dimensions tablet structure A: length (mm), width (mm), height (mm), average, standard deviation (stdev) and % relative standard deviation (%RSD). Table S3: Uniformity of mass tablet structure B: weight (mg), average weight, standard deviation (stdev), % relative standard deviation (%RSD) and % Deviation from average weight. Table S4: Uniformity of dimensions tablet structure B: length (mm), width (mm), height (mm), average, standard deviation (stdev) and % relative standard deviation (%RSD). Table S5: Uniformity of mass tablet structure C: weight (mg), average weight, standard deviation (stdev), % relative standard deviation (%RSD) and % Deviation from average weight. Table S6: Uniformity of dimensions tablet structure C: length (mm), width (mm), height (mm), average, standard deviation (stdev) and % relative standard deviation (%RSD). Table S7: Weibull model fit results: goodness of fit (r^2^), residual sum of squares (RSS), scale factor *kd* and shape factor *n* (release exponent), normalisation factor (NF) (%), estimated surface area to volume ratio (SA/V, [mm^−1^]) and number of pores for top and bottom of tablet (pores). Table S8: Hopfenberg model fit results: goodness of fit (r^2^), residual sum of squares (RSS), erosion rate constant *k*_0_, release exponent *n*, surface area to volume ratio (SA/V, [mm^−1^]), number of pores initially available to the dissolution medium.

## Glossary

EPSRC	Engineering and Physical Sciences Research Council (EPSRC)
CMAC	Center for Continuous Manufacturing and Advanced Crystallisation at the University of Strathclyde
HPLC	High-Performance Liquid Chromatography
Ph Eur	European Pharmacopoeia
USP	United States Pharmacopeia
