# Supplementary material for: An Additive Manufacturing MicroFactory: Overcoming Brittle Material Failure and Improving Product Performance through Tablet Micro-Structure Control for an Immediate Release Dose Form
Source: Polymers (Basel). 2024 Sep 11;16(18):2566. doi: 10.3390/polym16182566 (PMC11435212; doi:10.3390/polym16182566)
Supplement: Supplementary file 1 [file polymers-16-02566-s001.zip › polymers-3136029-supplementary.pdf]

Supplementary Information

1. Uniformity of mass and dimensions of printed tablets

1.1. Ellipse 22x12x5, no top/bottom layer, 47.3% Infill: ***Tablet A***

Tablets structure A	weight (mg)	% Difference from average
1	497.5	-0.18
2	497.0	-0.28
3	495.6	-0.56
4	494.1	-0.86
5	499.4	0.20
6	506.7	1.67
average	498.4	
stdev	4.4	
%RSD	0.9	

Table S 1: Uniformity of mass tablet structure A: weight (mg), average weight, standard deviation (stdev), % relative standard deviation (%RSD) and % Deviation from average weight.

Tablet A	length (mm)	width (mm)	height (mm)
1	21.77	11.78	4.8
2	21.75	11.78	4.75
3	21.72	11.77	4.8
4	21.37	11.76	4.77
5	21.72	11.8	4.81
6	21.79	11.76	4.77
average	21.69	11.78	4.78
stdev	0.16	0.02	0.02
%RSD	0.73	0.13	0.49

Table S 2: Uniformity of dimensions tablet structure A: length (mm), width (mm), height (mm), average, standard deviation (stdev) and % relative standard deviation (%RSD).

1.2. Ellipse 22x12x5, no top, 40.6% Infill: *Tablet B*

Tablets Micro-structure B	weight (mg)	% Difference from average
1	524.5	0.3
2	517.4	-1.0
3	522.2	-0.1
4	527.1	0.8
5	523.6	0.2
6	521.6	-0.2
average	522.7	
Stdev	3.3	
%RSD	0.6	

Table S 3: Uniformity of mass tablet structure B: weight (mg), average weight, standard deviation (stdev), % relative standard deviation (%RSD) and % Deviation from average weight.

Tablet B	length (mm)	width (mm)	height (mm)
1	21.97	11.89	4.88
2	21.96	11.92	4.92
3	21.94	11.92	4.81
4	21.96	11.88	4.85
5	21.9	11.86	4.76
6	21.91	11.83	4.75
average	21.94	11.88	4.83
stdev	0.03	0.04	0.07
%RSD	0.13	0.29	1.39

Table S 4: Uniformity of dimensions tablet structure B: length (mm), width (mm), height (mm), average, standard deviation (stdev) and % relative standard deviation (%RSD).

1.2. Ellipse 22x12x5, top/bottom layer, 35% Infill: ***Tablet C***

Tablet C	weight (mg)	% Deviation from average
1	519.1	0.0
2	520.8	0.3
3	524	0.9
4	523.1	0.7
5	507.6	-2.2
6	520.9	0.3
average	519.3	
stdev	6.0	
%RSD	1.1	

Table S 5: Uniformity of mass tablet structure C: weight (mg), average weight, standard deviation (stdev), % relative standard deviation (%RSD) and % Deviation from average weight.

Tablet C	length (mm)	width (mm)	height (mm)
1	21.86	11.86	4.82
2	21.89	11.87	4.82
3	21.87	11.84	4.89
4	21.89	11.87	4.89
5	21.92	11.89	4.77
6	21.93	11.99	4.81
average	21.89	11.89	4.83
stdev	0.03	0.05	0.05
%RSD	0.12	0.45	0.99

Table S 6: Uniformity of dimensions tablet structure C: length (mm), width (mm), height (mm), average, standard deviation (stdev) and % relative standard deviation (%RSD).

2. FTIR analysis

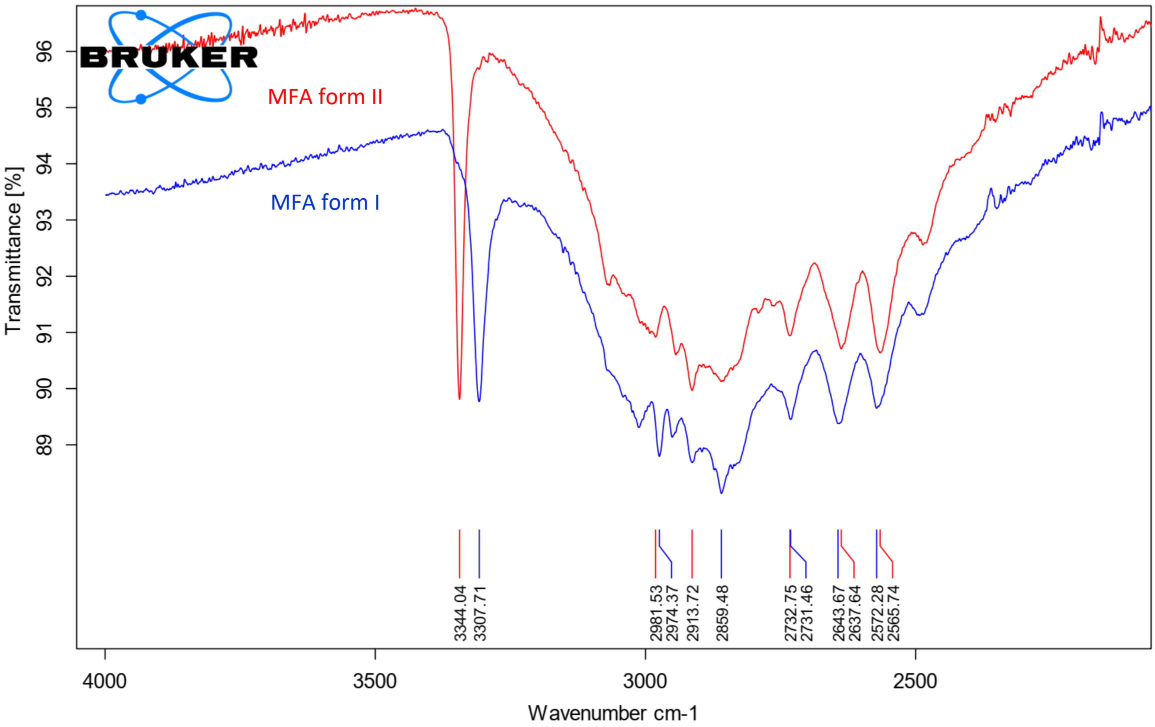

Figure S 1: FTIR spectra of MFA: Form I (blue) with N-H stretch at 3307 cm^{-1}, Form II (red) with N-H stretch at 3344 cm^{-1}.

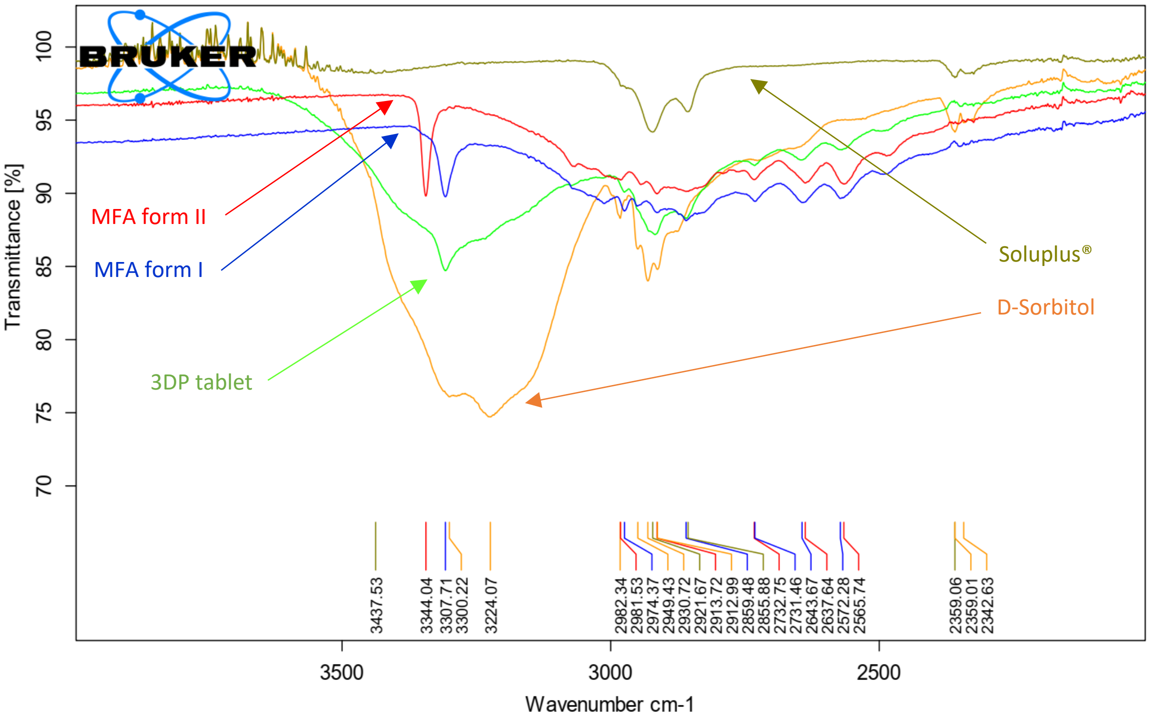

Figure S 2: FTIR spectra of MFA form I (blue), form II (red), 3DP tablet (green), Soluplus® (olive) and D-Sorbitol (orange).

3. Mathematical description of dissolution data

3.1. Weibull

Weibull	r^2	RSS	kd	n	NF (%)	SA/V (mm^{-1})	pores
Tablet A	1.0000	0.1	0.110	1.088	96.6	3.3	312
Tablet B	0.9997	4.4	0.008	1.811	92.8	3.5	138
Tablet C	0.9991	14.8	0.001	2.428	90.2	0.6	0

Table S 7: Weibull model fit results: goodness of fit (r^2), residual sum of squares (RSS), scale factor kd and shape factor n (release exponent), normalisation factor (NF) (%), estimated surface area to volume ratio (SA / V, [mm^{-1}]) and number of pores for top and bottom of tablet (pores).

3.2. Hopfenberg

Hopfenberg	r^2	RSS	k_0	n	SA / V (mm^{-1})	pores
Tablet A	0.9714	172.8	25.7	0.50	3.3	312
Tablet B	0.9969	12.9	4.8	0.58	3.5	138
Tablet C	0.9894	51.1	0.5	0.76	0.6	0

Table S 8: Hopfenberg model fit results: goodness of fit (r^2), residual sum of squares (RSS), erosion rate constant k_0, release exponent n, surface area to volume ratio (SA / V, [mm^{-1}]), number of pores initially available to the dissolution medium.
